# Water Quality and *Anopheles gambiae* Larval Tolerance to Pyrethroids in the Cities of Douala and Yaoundé (Cameroon)

**DOI:** 10.1155/2012/429817

**Published:** 2012-06-07

**Authors:** Billy Tene Fossog, Edmond Kopya, Cyrille Ndo, Benjamin Menze-Djantio, Carlo Costantini, Flaubert Njiokou, Parfait Awono-Ambene, Christophe Antonio-Nkondjio

**Affiliations:** ^1^Laboratoire de Recherche sur le Paludisme, Organisation de Coordination pour la Lutte Contre les Endémies en Afrique Centrale (OCEAC), P.O. Box 288, Yaoundé, Cameroon; ^2^Faculty of Sciences, University of Yaoundé I, P.O. Box 337, Yaoundé, Cameroon; ^3^Institut de Recherche pour le Développement (IRD), UR 016, 911 avenue Agropolis, P.O. Box 64501, 34394 Montpellier Cedex 5, France; ^4^Vector Group, Liverpool School of Tropical Medicine, Pembroke Place, Liverpool L3 5QA, UK

## Abstract

The poor management of the urban environment in sub-Saharan Africa is affecting *Anopheles gambiae* susceptibility to insecticides. A study was undertaken to assess the influence of breeding sites physicochemical parameters on malaria vectors population tolerance to insecticides. A total of 18, 262 larvae collected from 104 breeding sites were exposed to diagnostic concentrations of permethrin and deltamethrin. Larvae originating from cultivated sites were more tolerant than larvae from polluted or nonpolluted sites. No significant difference was observed between polluted and nonpolluted sites. Field larvae were 142 to 325 times and 6.08 to 9.57 times more tolerant to deltamethrin and permethrin, respectively, than larvae of the *A. gambiae* Kisumu strain used as control. A low but significant correlation was detected between physicochemical parameters and larval insecticide tolerance. Cultivated sites were negatively and significantly correlated to larval tolerance to both deltamethrin (*r* = −0.421; *P* < 0.0001) and permethrin (*r* = −0.392; *P* < 0.0001). Dissolved oxygen (*r* = +0.466; *P* < 0.0001) and ammonia (*r* = −0.205; *P* = 0.04) appeared significantly correlated to larval tolerance to deltamethrin. The data suggest a direct correlation between some characteristics from the breeding sites and larval tolerance to pyrethroids.

## 1. Introduction

The amount of pollutants released by domestic or industrial activities in the natural environment has increased during the past decades [1]. Most of these compounds accumulate in rivers or stagnant water bodies. Despite the high toxicity of some of these compounds, their impact on the aquatic fauna and the natural ecosystem is poorly understood. There is actually a fear that exposure of mosquito larvae to these substances could reduce their level of susceptibility to insecticides used for vector control. The rapid unplanned urbanization of Sub-Saharan Africa cities which had a great impact on natural ecosystems is considered to have favored the adaptation of anopheline to various xenobiotics and the expansion of their niche to polluted habitats [2, 3] and agricultural cultivated sites [4]. High prevalence of vector resistance to pyrethroids has been reported in a great number of cities across sub-Saharan Africa [5, 6]. Broadly two major mechanisms are responsible for the majority of insecticide resistance cases: reduced target site sensitivity due to mutations at the DNA level and metabolic detoxification through increased enzyme activities. Enzymes involved in insecticide metabolism are members of three large detoxification multigene families: the glutathione S transferases (GSTs), cytochrome P450s, and esterases. Reduced target site resistance is specific to insecticides with similar mode of action whereas, metabolic resistance has a broad spectrum and is nonspecific. Compounds responsible for the selection of vector resistance to insecticides are numerous and include a wide range of substances, such as, plants allelochemicals, heavy metals, chemical wastes derived from domestic or industrial discharges, oil spillage, and insecticides [2, 7, 8]. Understanding cross resistance of mosquitoes to insecticides and these xenobiotics could improve vector control strategies and the design of new diagnostic or control measures.

In the cities of Douala and Yaoundé, a low susceptibility of vectors to insecticides particularly DDT and permethrin was recorded [9]. However, the study could not draw any direct relation between the increase prevalence of resistance alleles (kdr gene) in mosquito populations and the source of selection (use of insecticides treated nets by the population, domestic, or industrial pollution). This study has as main objective to assess the correlation between breeding sites physicochemical characteristics and *A. gambiae* larvae susceptibility to sublethal concentration of permethrin and deltamethrin and identify parameters or substances selecting for mosquito resistance to pyrethroids.

## 2. Methods

### 2.1. Study Sites

The study took place in the cities of Yaoundé (3°51′ N 11°30′ E) and Douala (3°48′ N 10°08′ E) the two major urban cities in Cameroon. These cities are situated within the Congo-Guinean phytogeographic zone characterized by a typical equatorial climate with two rainy seasons extending from March to June and from September to November. Douala is situated near the Atlantic coast 1 m above sea level and receives over 3,500 mm of rainfall annually whereas, the annual average rainfall in Yaoundé is 1,700 mm. Yaoundé is located inland 250 km east of Douala. The city is situated 800 m above sea level and is surrounded by many hills.

Larval collections in the city of Yaoundé were carried out in nine districts: Mokolo, Messa, Olezoa, Ngousso, Gare, Nkolbisson, Dakar, Nkolondom, and Combattant. In Douala, field collections took place in seven districts: Ndogbong, Bonaberi, Bessengue, Nylon, PK15, Village, and Yassa.

The study was conducted under the ethical clearance N° 216/CNE/SE/09 delivered by the Cameroon National Ethics Committee Reference N° IORG0006538-IRB00007847-FWA00016054.

### 2.2. Breeding Sites Characteristics

Breeding places were classified in three categories after visual inspections: organically polluted, nonpolluted, and cultivated sites.

- “Polluted breeding sites” are semipermanent water collections containing domestic wastes or organic products in decomposition which could be invaded by moisture or alga (This make reference mainly to organic pollution which could be easily recognizable).
- “Nonpolluted breeding sites” are temporary water collections created after rains or resulting from a clean water source and mainly without any sign of organic pollution.
- “Cultivated breeding sites” are created by the practice of agriculture and include furrows and irrigation pits.

Measurements of physicochemical characteristics of breeding sites were recorded using a Wagtech portable Kit (CP1000). Parameters measured were temperature (°C), conductivity expressed in micro Siemens per cm (*μ*s/cm), dissolve Oxygen, phosphates, nitrates (NO_3_^−^), ammonia (NH_4_^+^), aluminium, iron, potassium, and total hardness concentrations all expressed in mg/L.

### 2.3. Mosquito Identification

Anopheline larvae were identified morphologically using the Gillies and Coetzee keys [10]. Mosquitoes belonging to the *Anopheles gambiae* complex were subjected to PCR assays designed for species and molecular forms identifications [11]. Genomic DNA used for molecular analysis was extracted from larvae according to Cornel [12] protocols.

### 2.4. Bioassay Experimentations

#### 2.4.1. Preparation of Test Solutions for Larvae Bioassays

Two technical grade compounds were used for the bioassays, permethrin 96.2% cis : trans 25 : 75 (Sigma-Aldrich Taufkirchen, Germany), and deltamethrin 98.3% (Sigma-Aldrich Taufkirchen, Germany). Stock solutions and serial dilutions were prepared following the protocol described in WHO guidelines [13]. A stock solution at 1% was prepared by mixing 200 mg of the technical grade material to 20 mL of absolute ethanol. This stock solution was then serially diluted in ethanol. Test concentrations were prepared by adding 10 to 1000 *μ*L of the appropriate dilution to 100 mL of distilled water.

#### 2.4.2. Bioassays with Larvae

Larvae of the *A. gambiae* kisumu strain (used as control) were exposed to a wide range of test concentrations to determine the range of toxicity of permethrin and deltamethrin. Then they were exposed to narrower range of concentrations (0.001 to 0.5 mg/L) leading to mortality rate comprised between 0 and 100%. For each experiment, 20–30 larvae per cup were exposed to different concentrations of permethrin and deltamethrin. A control was run as well. At least 4 different concentrations were tested and each experiment was repeated 4 times. The resulted mortality was used to determine the diagnostic lethal concentrations killing 30, 50, 80, and 99% of larvae which represented concentrations used to screen field populations. The Yaoundé laboratory colony and field collected *Anopheles gambiae* larvae, consisting of third and early fourth instars were exposed in 100 mL freshly prepared insecticide solution at the required concentration. Control cups had 99 mL of distilled water with 1 mL of absolute ethanol added. Batches of 20 to 30 larvae were distributed per cup. Larvae of each breeding site were exposed to all four diagnostic concentrations. After an initial observation period of 1 hour in distilled water, larvae were transferred into test cup with the required insecticide concentration. Larval mortality was recorded after 24 hours exposure. Larvae were considered dead when they were incapable of any active movement when touched. The mortality was corrected by the formula of Abbott [14] if it was between 5 and 20%.

The lethal concentration killing 50% and 90% of larvae (LC50 and LC90) was calculated. The ratio RR50 was determined to assess the level of tolerance of each population compared to the Kisumu strain. RR50 = LC50 assay/LC50 Kisumu strain. Arbitrarily a population was considered susceptible when RR50 was less than 2 potentially resistant when RR50 was between 2 and 5 and resistant when RR50 was over 5. The characteristics of breeding sites where originated larvae used for bioassays were recorded.

### 2.5. Statistical Analysis

The slope of the regression lines and the estimates of lethal concentrations killing 50% and 90% of field larvae was determined using the software R and WinDL 2 which uses the iterative method of maximum likelihood to fit a linear regression between the log of insecticide concentration and the probit of mortality.

The relationships between larval mortality to either permethrin or deltamethrin and physicochemical parameters were first tested with univariate analysis. *P* values <0.05 were considered significant. Then a multivariate regression analysis with a conditional stepwise procedure was conducted. All variables significantly associated with the dependent variable in univariate analysis, and variables with *P* values <0.25 were introduced into the model. The goodness of fit of the final model was assessed using the Hosmer and Lemeshow statistics. All these analyses were performed using the MedCalc V11.5.0.0. Percentages were compared using Pearson's chi-squared test or Fisher's exact test. Comparison between means was assessed using ANOVA or Kruskals Wallis test in case of inequality of sample variances. Canonical correlation analyses were carried to assess the level of correlation between variables. Prior to canonical analyses, all physicochemical and tolerance variables were square root transformed. Then a second level of analysis was carried to assess the level of correlation between physicochemical characteristics of breeding sites and larvae tolerance to permethrin or deltamethrin. The Bartlett test was applied to test for the statistical significance of the correlation. The proportion of variance explained by the correlation was assessed by a redundancy analysis. All these analyses were carried using the software R.

## 3. Results

### 3.1. Field Sampling and Breeding Sites Characteristics

Anopheline larvae were sampled from 104 breeding sites, 41 in Douala, and 63 in Yaoundé. Larval collections in Yaoundé were conducted in polluted, nonpolluted, and cultivated sites, whereas in Douala, larvae were sampled only from polluted and nonpolluted sites. The concentration of almost all parameters measured was high in polluted sites; however, only iron in breeding sites of both Douala and Yaoundé and potassium in Yaoundé were found significantly different (*P* < 0.05) (Table 1).

### 3.2. M and S Form Identification

A total of 346 larvae originating from 34 breeding sites in Yaoundé and 10 breeding sites in Douala were identified by PCR down to the molecular form level. Breeding sites were chosen in order to cover the different categories of habitats (polluted, nonpolluted, and cultivated) and almost all districts in each city. Of the 34 breeding sites analyzed in Yaoundé, 14 had exclusively the S form, 10 exclusively the M form while both forms were found in 10 breeding sites. The S form was predominant in breeding sites situated in cultivated areas (>90% of larvae) whereas the M form was abundant in polluted and nonpolluted sites (>90% of larvae) distributed in the city centre. Of the 97 larvae identified from 10 breeding sites in Douala, 3 were of the S molecular form and the remaining (*n* = 94) of the M molecular form. The three S form larvae were recorded from only 2 nonpolluted breeding sites in the same district.

### 3.3. Level of Tolerance of Field Larvae to Permethrin and Deltamethrin

**Figure 1:**
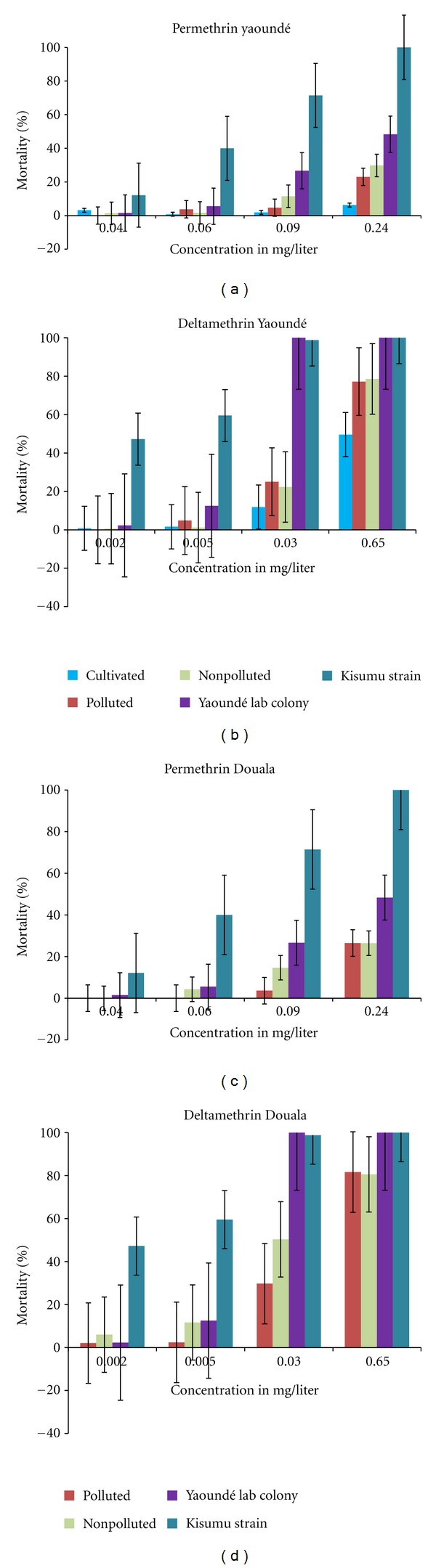
Diagrams showing mortality rates of third and fourth instars larvae after exposure to sublethal concentrations of permethrin and deltamethrin in Yaoundé ((a) and (b)) and Douala ((c) and (d)).

Diagnostic concentrations established after exposing the *A. gambiae* kisumu strain with mortality rates ranging between 10 and 100% were 0.04, 0.06, 0.09, and 0.24 mg/L for permethrin and 0.002, 0.005, 0.03, 0.65 mg/L for deltamethrin, respectively. A total of 18,262 late third instars or fourth instars larvae collected from the field were exposed to these diagnostic concentrations. Larvae collected from cultivated areas were more tolerant (low mortality) to both permethrin and deltamethrin than larvae originating from polluted or nonpolluted sites (*P* < 0.001). No significant difference was recorded for the mortality rate of larvae originating from polluted and nonpolluted sites in either Yaoundé (Figures 1(a) and 1(b)) or Douala (Figures 1(c) and 1(d)) (*P* > 0.05). The lethal concentration killing 50% of larvae (LC50) varied from 0.425 to 0.670 mg/L for permethrin and 0.284 to 0.651 mg/L for deltamethrin (Table 2).

The RR50 ratio for deltamethrin ranged from 142 for nonpolluted sites to 325.5 for cultivated sites, this ratio for permethrin varied from 6.08 for nonpolluted sites to 9.57 for cultivated sites suggesting high tolerance of field larvae to both molecules. Increased tolerance in comparison to the kisumu strain was also recorded with the Yaoundé laboratory colony with the RR50 ratio varying from 3.38 for permethrin to 4 for deltamethrin (Table 2).

### 3.4. Level of Association between Larval Mortality Rate to Either Permethrin or Deltamethrin and Physicochemical Characteristics of Breeding Sites

Multivariate analysis with larval mortality to sublethal concentration to either permethrin or deltamethrin as outcome variable and either physicochemical variables (conductivity, dissolve oxygen, nitrates, ammonia, potassium, iron, aluminium, zinc) and breeding sites characteristics (polluted, cultivated, and nonpolluted sites) as explanatory variables were performed. Cultivated sites appeared negatively and significantly correlated to mortality to both deltamethrin (*r* = −0.421; *P* < 0.0001) and permethrin (*r* = −0.392; *P* < 0.0001). Dissolved oxygen (*r* = +0.466; *P* < 0.0001) and Ammonia (*r* = −0.205; *P* = 0.04) appeared significantly correlated to mortality to deltamethrin (Table 3).

### 3.5. Canonical Correlation Analysis between Larval Tolerance to Both Permethrin and Deltamethrin and Breeding Sites Physicochemical Characteristics

The analysis included 57 breeding sites with larvae exposed to both permethrin and deltamethrin. The lethal concentration killing 50% or 90% of larvae was used as reference values for tolerance to either permethrin or deltamethrin. Tolerance to either permethrin or deltamethrin was found to be highly correlated. The level of correlation detected between physicochemical parameters and tolerance to deltamethrin was 0.623 and 0.44 for LC50 and LC90 values, respectively. For permethrin the levels of correlation recorded were 0.32 and 0.19 for LC50 an LC90 respectively. A low but significant correlation was recorded after testing with the Bartlett test for the significance of the correlation (*P* = 0.03). Redundancy analysis showed on their part that the present correlation explained just 20.04% of the total variance and this was not significant (*P* = 0.064).

## 4. Discussion

 Field larvae appeared more tolerant to permethrin than to deltamethrin. The data was consistent with recent findings in the area showing a large prevalence of resistance to permethrin [9, 15]. Although permethrin has never been used in the country for indoors residual spraying and less frequently for bed nets impregnation, it was largely affected by resistance. Selection against this molecule may have occurred as a result of cross resistance with DDT which was used during several decades for indoors residual spraying [16] and later as a consequence of high selective pressure of insecticides containing pyrethrins used in household (including bed nets) and in agriculture [15, 17].

High tolerance to both permethrin and deltamethrin was recorded with larvae originating from cultivated areas, whereas no significant difference was recorded for larvae originating from polluted and nonpolluted sites. The high tolerance of larvae originating from cultivated areas could result to their frequent exposure to pesticides used regularly in urban farming [15]. On the other hand, the absence of clear difference between mosquitoes originating from polluted and nonpolluted sites could indicate a low selective pressure in polluted breeding sites. The fact that, the study took place during the rainy season when semipermanent water collections are regularly filled by rainfall could have diluted the level of pollution of habitats characterized visually as polluted. However, the situation highlights the need to associate to visual description of breeding sites, measurable parameters which could guarantee a better classification of breeding sites to one or another category.

Although initial findings by Antonio-Nkondjio et al. [9] detected a high prevalence of the Kdr alleles in adult mosquito populations of the cities of Douala and Yaoundé and the strong correlation found between kdr allele presence and vector resistance to permethrin or DDT, it is not sure that tolerance to permethrin and deltamethrin observed during this study is mediated by this sole mechanism. Indeed biochemical assays undertaken on *A. gambiae* specimens resistant to permethrin from Yaoundé detected overproduction of mixed function oxidases [17]. Possibly tolerance to pyrethroids may involve additional resistant mechanisms. Studies conducted so far in Cameroon have unusually involved the use of biochemical or microarray tools; this could constitute a limit in understanding the real influence of metabolic detoxification mechanisms in vector resistance. Several detoxification mechanisms involving overproduction of P450s genes, redox genes, and cuticular precursor genes were reported in resistant *A. gambiae* populations from neighboring urban cities in Benin and Nigeria [18, 19].

A significant correlation was detected between some physicochemical parameters and larval insecticide tolerance. Parameters closely associated to vector tolerance to either permethrin or deltamethrin included cultivated sites, the concentration of dissolve oxygen and ammonia. The use of pesticides in agriculture has been pointed as one of the major factors driving insecticide resistance in malaria vectors [20, 21]. Ammonia lethal effect on mosquitoes is still not well documented. Yet from studies conducted on aquatic organisms, such as, juvenile shrimps, it has been reported that high concentration of ammonia may induce reduced growth, increased susceptibility to pathogens and high mortality in susceptible populations [22]. Possibly detoxification mechanisms alongside osmotic regulation mechanisms may be enhanced in resistant individuals as it has been reported for shrimps [23]. Whereas, tolerance to oxygen may promote increased level of expression of genes involved with the oxidative stress. It was demonstrated for *A. gambiae* that, oxidative stress genes, such as, oxidase resistance (OXR1) apart from protecting against oxidative stress also regulate the basal level of catalase and glutathione peroxidase expression, two enzymes involved in the detoxification of hydrogen peroxide, and several other xenobiotics [24].

Beside, the low correlation detected between breeding sites physicochemical characteristics and larval tolerance could be associated to the heterogeneous pattern of *A. gambiae* M and S forms susceptibility. Indeed the S form predominated in cultivated sites where mosquitoes larvae appeared more tolerant than in nonpolluted or polluted sites where the majority of the M form originated. This heterogeneity could have affected the trend of susceptibility detected during this study and the correlation with physicochemical parameters. It has previously been documented that kdr-based pyrethroid resistance in the West African region is more prevalent in the S form than in the M form [5, 25–27]. A comprehensive sampling design to discard the possible influence of molecular forms on vector tolerance to insecticide would have requested to compare pyrethroids susceptibility of the S form across different categories of breeding sites and similarly for the M molecular form. This sample design is not possible in the cities of Douala and Yaoundé since we detected a nonuniform distribution of both forms across the two cities. Nevertheless the study was able to identify some physicochemical parameters influencing larvae susceptibility to insecticides. Several xenobiotics, such as, heavy metals, petroleum products, regularly influence species biological responses and probably affect vectors susceptibility to insecticides. There is a need to assess the influence of these pollutants on the increase vector tolerance to insecticides.

## 5. Conclusion

Despite the expansion of vector resistance across sub-Saharan Africa, there is not still sufficient information on the role of xenobiotics selection on vector resistance to insecticides. This information could be particularly important for a good management of insecticide resistance.

## Figures and Tables

**Table 1:** Physicochemical characteristics of breeding sites sampled in Douala and Yaoundé.

	Yaoundé							Douala				
Parameters	Cultivated		Nonpolluted		Polluted		*P* value	Nonpolluted		Polluted		*P* value
	*N*	Mean (SE)	*N*	Mean (SE)	*N*	Mean (SE)		*N*	Mean (SE)	*N*	Mean (SE)	
Aluminium	17	0.09 (0.06)	24	0.06 (0.01)	22	0.07 (0.02)	0.35	22	0.07 (0.03)	18	0.03 (0.01)	0.21
Ammonia	17	1.81 (0.73)	24	1.16 (0.29)	22	3.04 (1.01)	0.09	22	0.58 (0.26)	19	0.66 (0.21)	0.82
Conductivity	17	480.14 (73.35)	24	502.90 (64.30)	22	1027.87 (183.86)	**0.002**	22	334.34 (58.11)	19	657.81 (175.09)	0.11
Iron	17	3.44 (0.46)	24	6.75 (0.40)	22	9.46 (1.32)	**0.000**	22	3.91 (0.42)	19	5.62 (0.65)	**0.03**
Nitrates	17	5.25 (1.49)	24	6.41 (1.43)	22	6.88 (2.36)	0.408	22	4.73 (1.40)	19	5.06 (1.13)	0.86
Dissolve oxygen	15	6.80 (1.02)	18	6.93 (0.59)	7	5.89 (1.57)	0.786	19	135.36 (12.55)	19	98.08 (15.33)	0.07
Phosphates	2	0.57 (0.25)	6	1.56 (0.96)	19	2.16 (0.72)	0.72	22	8.94 (2.55)	19	29.71 (8.43)	0.06
Potassium	17	17.17 (3.09)	24	22.52 (3.38)	22	51.94 (12)	**0.000**	16	5.71 (1.20)	18	9.34 (1.60)	0.08
Temperature	15	31.47 (0.85)	18	29.35 (0.59)	7	31.03 (1.01)	0.102	22	32.96 (0.50)	19	32.27 (0.72)	0.43
Total hardness	17	76.77 (13.91)	24	86.67 (13.02)	22	118.86 (21.64)	0.5	—	—	—	—	—

*N*: sample size, mean: average concentration (in mg/L) of physicochemical parameters in breeding sites, SE: standard error, *P* value significant if *P* < 0.05, in bold significant values.

**Table 2:** Susceptibility of *A. gambiae* larvae originating from different types of breeding sites to permethrin and deltamethrin.

	Deltamethrin					Permethrin				
Sites	*N*	Regression line	LC50 (SE)	LC90 (SE)	RR50	*N*	Regression line	LC50 (SE)	LC90 (SE)	RR50
Kisumu strain	272	*Y* = 137*X* − 0.34	0.002 (0.001)	0.02 (0.003)	1	560	*Y* = 54.8*X* − 3.9	0.07 (0.002)	0.11 (0.005)	1
Yaoundé lab colony	516	*Y* = 605.4*X* − 4.97	0.008 (0.001)	0.01 (0.003)	4	636	*Y* = 11.96*X* − 2.8	0.24 (0.01)	0.42 (0.03)	3.38
Yaoundé										
Cultivated	1713	*Y* = 4.6*X* − 2.99	0.65 (0.02)	1.13 (0.04)	325.5	1766	*Y* = 6.4*X* − 4.31	0.67 (0.07)	1.01 (0.12)	9.57
Polluted	925	*Y* = 4.7*X* − 1.82	0.38 (0.02)	0.86 (0.04)	193.5	1013	*Y* = 5.8*X* − 3	0.52 (0.02)	0.89 (0.05)	7.36
Nonpolluted	3396	*Y* = 5.6*X* − 2.29	0.41 (0.01)	0.81 (0.02)	205,5	3451	*Y* = 6.96*X* − 2.97	0.43 (0.01)	0.74 (0.03)	6.08
Douala										
Polluted	1300	*Y* = 5.5*X* − 2.02	0.37 (0.02)	0.77 (0.03)	184.5	878	*Y* = 6.2*X* − 3.3	0.533 (0.03)	0.89 (0.06)	7.61
Nonpolluted	1581	*Y* = 4.06*X* − 1.15	0.28 (0.02)	0.83 (0.04)	142	2239	*Y* = 4.7*X* − 2.5	0.527 (0.02)	0.996 (0.05)	7.53

Sites: refer to the origin of mosquitoes used for bioassays, *N*: total number of *A. gambiae* larvae exposed to each insecticide, lethal concentrations killing 50% or 90% of larvae (LC50 and LC90) expressed in mg/liter, SE: standard error, RR50 = LC50 assay/LC50 Kisumu strain. A population is susceptible when RR50 < 2, potentially resistant when 2 < RR50 < 5, resistant when RR50 > 5.

**Table 3:** Correlation between breeding sites physicochemical characteristics and larval mortality to either deltamethrin or permethrin.

	Deltamethrin				Permethrin			
Parameters	*N*	Mean (95% CI)	*R*	*P* value	*N*	Mean (95% CI)	*R*	*P* value
Physicochemical characteristics								
Conductivity	104	602.9 (485.5–720.3)	−0.078	NS	98	602.68 (479.3–726)	+0.065	NS
Temperature	81	31.55 (30.9–32.2)	+0.027	NS	75	31.86 (31.06–32.7)	+0.172	NS
Dissolve oxygen	78	60.3 (44.4–76.2)	+0.466	<0.0001	73	59.77 (43.2–76.4)	+0.187	NS
Total hardness	63	95.24 (75.5–115)	+0.151	NS	63	95.24 (75.5–115.02)	+0.204	NS
Nitrates	104	5.72 (4.27–7.17)	−0.072	NS	98	6.07 (4.53–7.62)	−0.06	NS
Ammonia	104	1.45 (0.91–2)	−0.205	0.04	98	1.55 (0.98–2.12)	−0.084	NS
Aluminium	103	0.063 (0.04–0.09)	−0.069	NS	98	0.065 (0.04–0.09)	−0.053	NS
Potassium	97	23.03 (16.4–29.7)	−0.156	NS	93	24.60 (17.8–31.5)	−0.0098	NS
Iron	104	5.97 (5.19–6.76)	−0.026	NS	98	6.10 (5.28–6.92)	+0.028	NS
Phosphates	68	11.95 (6.30–17.60)	+0.087	NS	62	14.25 (7.05–21.44)	−0.23	NS
Physical characteristics								
Cultivated sites	17	—	−0.421	<0.0001	17	—	−0.392	<0.0001
Polluted sites	40	—	+0.113	NS	35	—	0.162	NS
Nonpolluted site	46	—	+0.203	NS	46	—	0.172	NS

*N*: sample size, mean: average concentration (in mg/L) of physicochemical parameters in breeding sites, 95% CI: Confidence interval at 95%, *R*: Correlation coefficient, NS: Nonsignificant, *P* value significant if <0.05.
